# Motivators and Barriers to Living Donor Kidney Transplant as Perceived by Past and Potential Donors

**DOI:** 10.1177/20543581221137179

**Published:** 2022-11-19

**Authors:** Julia Zazoulina, Keesha Khehra, Jagbir Gill

**Affiliations:** 1 The University of British Columbia, Vancouver, Canada

**Keywords:** kidney transplant, donors, motivators, barriers

## Abstract

**Background:**

For patients with end-stage kidney disease, living donor kidney transplant is the treatment of choice due to improved patient outcomes, longer graft survival, and reduced expenses compared with other forms of renal replacement therapy. However, organ shortage remains a challenge, and living donation rates have stagnated in recent years, particularly among men.

**Objective:**

To understand the motivators and barriers for past and potential living kidney transplant donors and inform policy and practice changes that support donors in the future.

**Methods:**

Past and potential living donors in British Columbia, Canada in the preceding 2 years were surveyed. Motivators and barriers were examined in 5 categories: family pressures and domestic responsibilities, finances, the recovery process, complications, and the transplant evaluation process. Participants ranked statements in each category on a Likert-type scale.

**Results:**

A total of 138 responses were collected. In both women and men, policies that address family and domestic responsibilities and finances were most strongly identified as motivators to donate. A large proportion of women and men reported that guaranteed job security (47% women and 38% of men), paid time off (51% of women and 42% of men), reimbursement of lost wages (49% of women and 38% of men), and protections to guarantee no impact on future insurability (62% of women and 52% of men) were significant motivators to donate. Timely and efficient medical evaluation was considered to be an important motivator for donation, with 52% of men and 43% of women reporting support for a “fast-track” option for evaluation to allow for a more rapid evaluation process. Median barrier and motivator scores were similar between women and men.

**Conclusion:**

Policies to decrease financial burden, ensure job security, improve childcare support, and offer a fast-track medical evaluation may motivate potential living kidney donors, irrespective of gender.

## Abrégé

**Contexte:**

La transplantation rénale avec donneur vivant est le traitement de choix pour les patients atteints d’insuffisance rénale terminale, car elle améliore les résultats de santé du patient, prolonge la survie du greffon et réduit les dépenses de santé comparativement à d’autres formes de thérapie de remplacement rénal. Cependant, la pénurie d’organes demeure un défi et les taux de dons vivants, particulièrement chez les hommes, ont stagné dans les dernières années.

**Objectifs:**

Comprendre ce qui motive les donneurs vivants d’un rein passés et potentiels, et connaître les obstacles qu’ils rencontrent. Informer les changements de politiques et de pratiques pour soutenir les donneurs dans l’avenir.

**Méthodologie:**

Un sondage mené auprès de personnes enregistrées en Colombie-Britannique (Canada) comme donneurs vivants passés et potentiels au cours des deux années précédant l’étude. Les facteurs de motivation et les obstacles ont été examinés selon cinq catégories: les pressions familiales et les responsabilités domestiques, les finances, le processus de rétablissement, les complications et le processus d’évaluation de la transplantation. Les participants devaient classer des énoncés dans chaque catégorie sur une échelle de Likert.

**Résultats:**

En tout, 138 réponses ont été recueillies. L’aspect financier et les politiques traitant des responsabilités familiales et domestiques ont été les éléments les plus fortement cités comme facteurs de motivation à donner, tant chez les femmes que chez les hommes. Une grande proportion de femmes et d’hommes ont déclaré que la sécurité d’emploi (F: 47 %; H: 38 %), les congés payés (F: 51 %; H: 42 %), le remboursement des pertes de salaire (F: 49 %; H: 38 %) et les protections visant à ne garantir aucun impact sur l’assurabilité future (F: 62 %; H: 52 %) étaient d’importants facteurs de motivation à donner. Une évaluation médicale rapide et efficace a également été considérée comme un important facteur de motivation au don, 52 % des hommes et 43 % des femmes ayant déclaré être en faveur d’une option « rapide » qui permettrait d’accélérer le processus d’évaluation. Les scores médians obtenus pour les défis et facteurs de motivation étaient semblables pour les femmes et les hommes.

**Conclusion:**

Les politiques visant à réduire le fardeau financier, à assurer la sécurité d’emploi, à bonifier le soutien pour la garde des enfants et à accélérer l’évaluation médicale sont susceptibles de motiver les potentiels donneurs vivants d’un rein, quel que soit leur sexe.

## Introduction

End-stage kidney disease (ESKD) affects more than 40 000 Canadians, with the incidence increasing each year.^1^ Although most ESKD patients receive renal replacement therapy (RRT) through dialysis, living donor kidney transplant (LDKT) remains the preferred treatment for ESKD. Recipients of LDKT have significantly better outcomes including improved graft survival and patient survival as compared to deceased donor kidney transplant recipients.^2^ Annual dialysis costs can be as high as $89 000 CDN per year of therapy,^3^ and even the most cost-effective home dialysis methods average around $40 000 per year.^4^ In comparison, transplant patients with a functioning graft incur roughly $28,000 annually after the first year.^3^ Increasing the living donor pool is both impactful to patient quality of life and cost-effective.^5^

The number of LDKT has remained low and relatively stable over the past decade. Only 492 of 1357 kidney transplants in Canada were from living donors in 2019, which is marginally above the 409 living donors in 2011.^1^ Over the same time frame, deceased donor kidney transplants rose from 572 to 938 annually in Canada.^1^ Thus, despite the increasing number of ESKD patients, and overall increase in transplants, there has not been a proportional increase in LDKTs.^1^

Not all potential donors proceed with donation. Men specifically are underrepresented in the donor population. Several reasons for this have been postulated, such as gender differences in financial security, family pressures, and altruism.^6-9^ Numerous barriers exist in the donation process, including health-related concerns, insufficient knowledge about kidney transplant, social circumstances, financial ramifications, and a rigorous screening process.^10-12^ While many countries around the world have instituted more comprehensive remuneration strategies leading to increased LDKTs,^13-18^ factors such as gender, culture, or education were not taken into account. There is little research on what actually matters most to potential donors and what supports assist in proceeding with donation.

The objective of this study was to characterize the motivating and deterring factors potential donors face in British Columbia (BC), Canada, to what degree each factor contributes to their willingness to donate, and whether these differ between women and men.

## Methods

### Participant Population

Individuals who had come forward as potential living donors at the Vancouver General Hospital or St. Paul’s Hospital transplant programs between January 2018 and December 2019 were invited to participate, regardless of the whether they ultimately donated or not. Participants were required to be at least 18 years of age, residents of BC, and could not have a diagnosis of kidney disease. The Research Ethics Board of the University of British Columbia—Providence Health Care approved this study (H18-02161).

### Survey Development

A *s*urvey was developed based on existing questionnaires used in previous studies on living donation.^9^ Detailed demographic information and participants’ familiarity with LDKT was captured because it has been suggested that individuals with lesser understanding of LDKT tend to overestimate associated risks, and previous evidence has shown that education on organ donation is correlated with higher donation rates.^19-21^ A modified version of the Living Donor Kidney Transplant Knowledge questionnaire developed by Rodrigue et al^22^ was used. Since the original questionnaire was targeted toward transplant recipients, it was modified to reflect the donor perspective.^22^ It consists of 14 true/false statements, and each participant was awarded a knowledge score out of 14. As charitable giving has been linked to higher Empathy Quotient (EQ) Scores, we included the EQ-8 scale.^23^ This is an abbreviated version of the original 60 item EQ questionnaire and has been found to be reliable and valid in comparison.^23^

Specific statements relating to potential barriers and motivators within 5 distinct domains were developed: (1) decision-making and evaluation process; (2) family pressures and domestic responsibilities; (3) finances and job security; (4) recovery process; and (5) complications. Participants were asked to state their level of agreement with each statement on a 5-point Likert-type scale (*strongly disagree, disagree, neutral, agree*, and *strongly agree*).

### Data Collection

Paper copies of the survey were sent via mail-out, and a link for online completion was also provided. The electronic version of the survey was hosted on the Qualtrics platform (Qualtrics, Provo, UT; Seattle, Washington; United States of America). No personal identifying information was asked in the survey. Each survey was linked with a participant code which was saved in a secured file. A letter preceded the survey stating that informed consent was acknowledged if the survey was completed and returned. Surveys that were returned by mail were inputted into Qualtrics by research team members.

### Data Analysis

Characteristics of participants were described overall and for women and men separately using medians and quartiles for continuous variables, or frequencies and proportions for categorical variables. The frequency of missing data was low (0%-5%) and was not differentially missing between women and men. The knowledge questionnaire is scored as a total number of correct responses, with a maximum score of 14. The EQ-8 Score was calculated per the scale’s scoring system,^23^ and the median score was compared between women and men.

Responses for barriers and motivators within each of the 5 domains were summarized as a mean quantitative score indicating the level of agreement reported for each potential barrier or motivator. Individual Likert-type scale responses were quantified using the following point system: *Strongly disagree* = 1, *Disagree* = 2, *Neutral* = 3, *Agree* = 4, *Strongly Agree* = 5, *N/A* = 0. Median scores for each domain were calculated by combining scores for each question and then calculating an average.

## Results

### Participant Demographics

A total of 138 out of 600 (23%) individuals responded to the survey, 128 (93%) of whom were either currently being assessed to be a living donor or had already been assessed but did not proceed with donation (46% each). Thirty-six percent of these participants reported not moving forward due to medical unsuitability and another 23% reported another donor came forward instead. The remaining 7% of participants had already donated a kidney.

Thirty-five percent of participants were male, and 65% were female. Male participants tended to be older with 48% of men aged above 60 years, whereas only 29% of women were older than 60 years. Men and women who responded reported similar sociodemographic characteristics in terms of race, country of birth, relationship status, and sexual orientation. Similar proportions of men and women reported having children (Table 1).

**Table 1.** Study Cohort Demographics.

	Total (N =	Gender	
Demographic		Male (N =	Female (N =
Age group			
18–30	9 (6.5%)	4 (8.3%)	5 (5.6%)
31–40	15 (10.9%)	3 (6.3%)	12 (13.3%)
41–50	29 (21.0%)	5 (10.4%)	24 (26.7%)
51–60	34 (24.6%)	12 (25%)	22 (24.4%)
>60	49 (35.5%)	23 (47.9%)	26 (28.9%)
Race			
Caucasian	126 (91.3%)	43 (89.6%)	83 (92.2%)
Indigenous	3 (2.2%)	1 (2.1%)	2 (2.2%)
Asian	6 (4.3%)	3 (6.3%)	3 (3.3%)
Black	1 (0.7%)	0	1 (1.1%)
Middle Eastern	2 (1.4%)	1 (2.1%)	1 (1.1%)
Born in Canada			
Yes	117 (85.4%)	40 (83.3%)	77 (86.5%)
No	20 (14.6%)	8 (16.7%)	12 (13.5%)
Relationship status			
In a relationship	106 (76.8%)	41 (85.4%)	65 (72.2%)
Single	18 (13.0%)	4 (8.3%)	14 (15.6%)
Divorced/separated	13 (9.4%)	3 (6.3%)	10 (11.1%)
Widowed	1 (0.7%)	—	1 (1.1%)
Sexual orientation			
Heterosexual	130 (94.2%)	44 (91.7%)	86 (95.6%)
LGBTQ	6 (4.3%)	3 (6.3%)	3 (3.3%)
Education			
Less than high school	3 (2.2%)	1 (2.1%)	2 (2.2%)
High school diploma	15 (10.9%)	9 (18.8%)	6 (6.7%)
Some postsecondary	58 (42.3%)	22 (45.8%)	36 (40%)
Bachelor’s degree	32 (23.4%)	7 (14.6%)	25 (27.8%)
Postgraduate degree^a^	29 (21.2%)	9 (18.8%)	20 (22.2%)
Current employment			
Working	93 (67.4%)	28 (58.3%)	65 (72.2%)
Retired	36 (26.1%)	18 (37.5%)	18 (20%)
Other^b^	9 (6.5%)	2 (4.2%)	7 (7.8%)
Children			
Yes	96 (69.6%)	32 (66.7%)	64 (71.1%)
No	42 (30.4%)	16 (33.3%)	26 (28.9%)
Personal income			
<15K	34 (24.6%)	3 (6.3%)	8 (8.9%)
16–30K	19 (13.8%)	5 (10.4%)	14 (15.6%)
31–50K	32 (23.2%)	8 (16.7%)	24(26.7%)
51–75K	35 (25.4%)	18 (37.5%)	17 (18.9%)
76–90K	17 (12.3%)	4 (8.3%)	13 (14.4%)
>90K	23 (16.7)	10 (20.8%)	13 (14.4%)
Household income			
<15K	2 (1.4%)	1 (2.1%)	1 (1.1%)
16–30K	5 (3.6%)	1 (2.1%)	4 (4.4%)
31–50K	23 (16.7%)	6 (12.5%)	17 (18.9%)
51–75K	34 (24.6%)	14 (29.2%)	20 (22.2%)
76–90K	21 (15.2%)	9 (18.8%)	12 (13.3%)
>90K	50 (36.2%)	17 (35.4%)	33 (36.7%)
Donation status			
Potential donors			

Abbreviation: LGBTQ = lesbian, gay, bisexual, transgender, and questioning (or queer).

a Includes masters, professional degree, and doctorate.

b Includes unemployed and looking for work, unemployed and not looking for work, and other.

**Figure 1.**
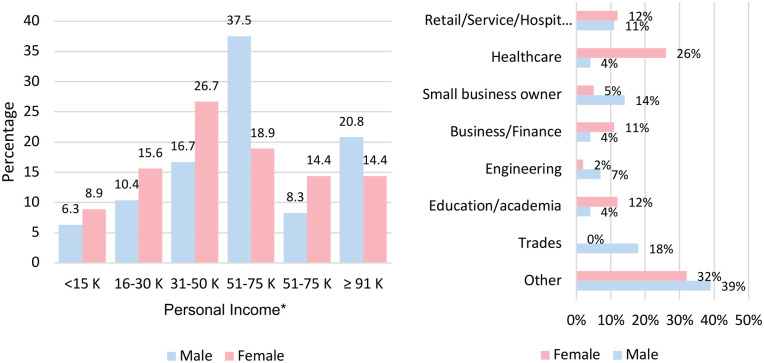
Personal income and employment industry. *Excluded individuals with no response for personal income (N = 1).

Figure 1 outlines the personal income and employment history in respondents. Sixty-seven percent of men reported earning a personal income of at least $50 000 annually, compared to 48% of women. Women were more likely to be employed than men (72% vs. 58% respectively), though the male cohort included more retired individuals. Half of female respondents held at least a bachelor’s degree versus 1 in every 3 males. Fifty-four percent of participants overall reported that they felt secure in their jobs. However, 14% of women reported their job situation as “very insecure,” while no men reported their job as “very insecure.”

The average EQ score was 10.54, with women having a higher score than men (11.74 vs 8.29).

### Knowledge of LDKT Process

The average knowledge score of participants was 11.5 out of 14. Mean overall knowledge scores were similar in men and women (11.7 vs 11.4 respectively). However, a slightly higher percentage of women (77%) compared to men (67%) knew that an LDKT lasts longer than a deceased donor transplant. Close to 60% of participants incorrectly thought the public health insurance plan in the province reimbursed indirect costs, and 47% did not know that a living donor would be given special priority to receive a kidney, should the need arise in the future (See Supplemental Figure 1).

### Motivators and Barriers

**Figure 2.**
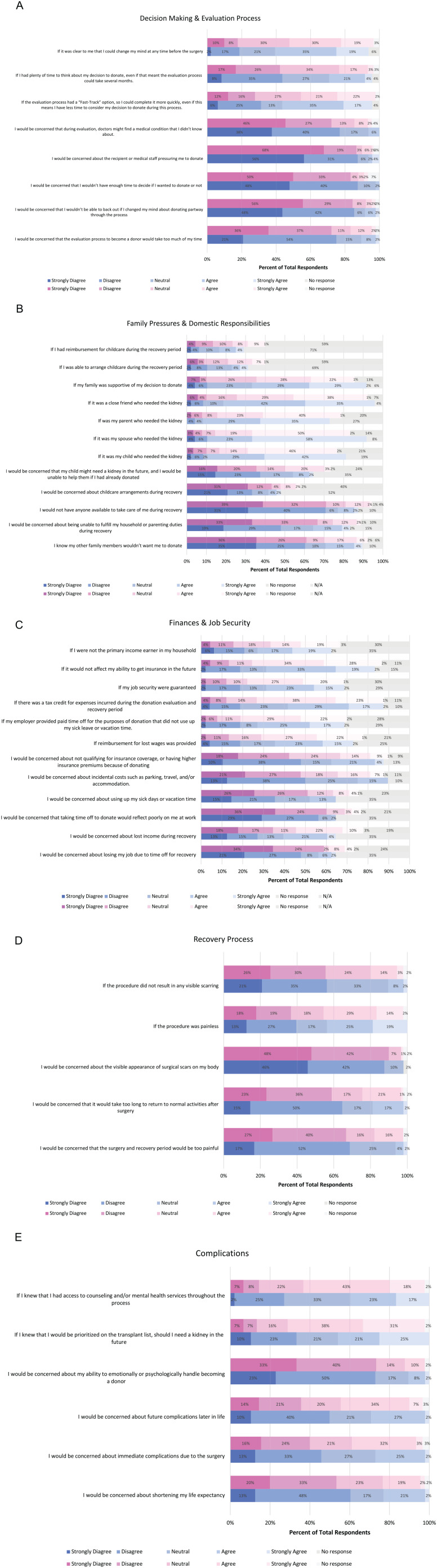
(A) Motivators and barriers in relation to the decision-making and evaluation process. (B) Motivators and barriers in relation to family pressures and domestic responsibilities. (C) Motivators and barriers in relation to finances and job security. (D) Motivators and barriers in relation to the recovery process. (E) Motivators and barriers in relation to complications. *Note.* Pink-shaded bars represent female respondents and blue-shaded bars represent male respondents.

Figure 2 A to E reports participant responses by women and men about motivators and barriers within each of the following domains: (1) decision-making and evaluation process; (2) family pressures and domestic responsibilities; (3) finances and job security; (4) recovery process; and (5) complications.

#### Decision-making and evaluation process

Most participants were not concerned about the identification of unknown medical conditions, coercion by medical staff, insufficient time to make a decision about donation, and not being able to have the freedom to change their mind. Timely and efficient medical evaluation was considered to be an important motivator for donation, with 52% of men and 43% of women reporting support for a “fast-track” option for evaluation to allow for a more rapid evaluation process. However, only 14% of women and 10% of men agreed or strongly agreed that delays in the evaluation process would be considered a barrier that would reduce their motivation to donate.

#### Family pressures and domestic responsibilities

The nature of the relationship to the recipient appeared to be more important to men surveyed, with more men reporting a high motivation to donate if the recipient was their spouse or a friend, and similar proportions of women and men were motivated to donate to their children or parents. A higher proportion of women reported concerns about household and parenting duties, with 22% of women and 10% of men reporting concern about their ability to maintain household or parenting duties during their recovery. Similarly, 19% of women and 12% of men felt that reimbursement for childcare expenses was an important motivator for donation.

#### Finances and job security

Concerns around financial and job insecurity were common in both women and men. Thirty-two percent of women and 25% of men reported concerns about lost income during recovery, with 12% of women and 8% of men reporting concern that they may lose their job due to time off for recovery. Similarly, 23% of women and 25% of men reported concerns about their ability to retain life insurance coverage or pay higher insurance premiums after donation. A large proportion of women and men reported that guaranteed job security (47% women and 38% of men), paid time off (51% of women and 42% of men), reimbursement of lost wages (49% of women and 38% of men), and protections to guarantee no impact on future insurability (62% of women and 52% of men) were significant motivators to donate. In addition, 61% of women and 46% of men supported a tax credit for expenses incurred during donation. Importantly, 32% of women and 36% of men reported that not being the primary household income earner was a motivator for donation, suggesting that financial concerns may be more significant for primary income earners.

#### Recovery process and complications

Similar proportions of women and men reported concerns about the impact of donation on their life expectancy and their ability to return to normal activities. Higher proportions of women were concerned about immediate postoperative complications (35% of women vs 27% of men), pain during recovery (18% of women vs 6% of men), scars (8% of women versus 0% of men), and long-term complications (41% of women vs 29% of men). Large proportions of both women and men reported that access to mental health and counseling resources and prioritization for kidney transplantation in the event of kidney failure for donors were strong motivators for donation.

**Figure 3.**
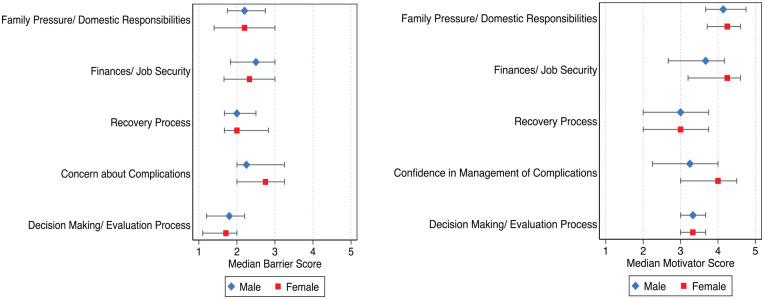
Median barrier and motivator scores.

Figure 3 summarizes the responses of women and men to questions and statements on barriers and motivators to living donation within the five domains. Median scores of Likert values are reported for barriers and motivators in each domain for women and men. Overall, median barrier scores were similar across the five domains studied, but median scores were slightly higher for barriers relating to concerns about complications, particularly amongst women. Motivators related to familial and domestic responsibilities and financial and job security were most commonly reported and were slightly more common amongst women.

## Discussion

Living kidney donation is critical to ensuring ESKD patients have timely access to transplantation with optimal posttransplant outcomes. This survey study describes the perceived motivators and barriers of potential living kidney donors among women and men who have come forward to donate a kidney in BC, Canada.

In both women and men, policies that address family and domestic responsibilities and finances were most strongly identified as motivators to donate. Concern around childcare expenses was highlighted by a larger percentage of women, with 42% reporting they would like more support for this. The magnitude of childcare expenses have not been extensively described in the literature, but the median total out of pocket expenses for a living donor have been estimated to be $1254, ranging from $531 to $2589.^24^ Programs such as the National Living Donor Assistance Center in the United States and the Living Organ Donor Reimbursement Programs across Canada typically provide some coverage for childcare, but the limits and caps surrounding this vary.^25,26^ Ensuring that prospective donors are broadly aware of the existing reimbursement options around childcare for donors may help motivate prospective donors to pursue donation.

Multiple studies have highlighted finances as a significant barrier to donation.^3,6,10,12,19,24^ We previously reported on the relationship between median household income and living donation rates in the United States, highlighting the potential impact of financial barriers on living donation.^6,27^ The findings of this study reiterate the ongoing financial concerns among potential donors despite numerous initiatives to combat this issue. Siddique et al^18^ looked at 23 countries and found that reimbursement programs increased donation numbers. However, it is possible that existing reimbursement strategies may be further improved.^13,28,29^ One study found median lost income for donors who experience unpaid time off work is approximately $5500, and about $2200 for out-of-pocket expenses and lost productivity.^24^ Median total cost exceeded $1000 for ~5% of donors, $5500 for 25% of donors, and in 13% of cases exceeded $10 000.^24^

In British Columbia, the maximum weekly income reimbursement through the Living Organ Donor Reimbursement Program is $543, and up to approximately $4000 for accommodation, travel, parking, and meals (mostly reserved for individuals traveling to the transplant program for work-up and surgery).^25^ The currently available reimbursement may fall short in many cases and may be a significant deterrent to organ donation. Nearly half of those surveyed indicated that financial reimbursement of lost wages and the assurance of job security would increase their motivation and ability to donate. This was particularly important if the prospective donor was the primary household earner, irrespective of gender.

This also confirms findings from prior studies that a lack of job security is an important factor for potential donors.^6,10,30^ Importantly, a significant proportion of women surveyed in this study worked in occupations which may offer more job security (such as health care and education). This may suggest that concerns of job security may be even further amplified among women who work in occupations with less stability.^25,31^ Job security is protected for Canadians who qualify for employment insurance (EI) during medical leaves of absence, but there are limitations in length of time covered.^32^ The proposed Living Donor Protection Act in the United States would ensure job security by allowing living donors to qualify for coverage under the Family and Medical Leave Act and also provides protection against discriminatory insurance policies against past living donors.^25,31^ Advancement of such policies is critical to motivate potential living donors.

Perceived delays in donor evaluation were identified as an important barrier to donation. The lengthy donor evaluation process has been noted to be a barrier by Canadian focus group participants in the past.^12^ In our study, approximately half of participants endorsed that they would prefer a Fast-Track option. This would allow donors to undergo work-up more rapidly, and minimize time off work, travel, and lost income during the process. While efforts have been made to define an ideal duration for efficient donor evaluation,^33^ it is important to acknowledge that individual circumstances for potential donors, particularly when potential donors are not yet fully committed to donation, may vary, thus impacting the expediency of the evaluation process. Many participants also endorsed that availability of additional mental health counseling through the transplant process would be a potential motivator for donation. Although, several studies have reported low rates of depression and anxiety among living donors,^34,35^ it is important to acknowledge there may be underreporting of mental health issues among respondents.^36^ A psychosocial evaluation is a standard part of most transplant programs; however, there are very few established mental health services available, and patients are often directed toward private counseling services when the need arises. Access to some degree of ongoing counseling or psychosocial support during and immediately following the transplant process could serve many potential donors.

Importantly, participants surveyed had inconsistent knowledge about key aspects of the donation process, such as the availability and nature of expense reimbursement programs and policies that ensure donor prioritization for transplant in the future. Thus, ensuring that the prospective donors have early and reliable access to information on policies and supports available for donors may be key to allow prospective donors to make informed decisions about living kidney donation.

Overall, there were few differences noted in perceived barriers between women and men in this study. This is surprising given that living kidney donation rates are consistently higher among women compared to men.^21^ Notably, the EQ was higher among women surveyed in this study, as has been seen among women in the general population as well.^23^ Higher empathy scores have been shown to be related to increased charitable giving, even when considering other sociodemographic factors.^23^ However, it is important to note that this survey was conducted amongst individuals that have already self-identified as potential donors and, therefore, the findings may underestimate the concerns that may preclude someone from considering donation. Further evaluation of a general population where donation may not have yet been considered may be useful to determine if there are indeed gender-specific barriers that contribute to the differences in donation rates between women and men. Future studies should also further evaluate the impact of donor and recipient incompatibility and willingness to consider kidney paired donation as a function of gender.

There are number of important limitations when interpreting the results of this study. Nonresponse bias is inherent to the study design of a voluntary mail-out survey. To maintain anonymity of the survey responders, we were unable to compare demographic characteristics of responders from nonresponders, further limiting the generalizability of the findings. Recall bias is also a risk of a survey study. To minimize this effect, we focused on individuals who were linked to the transplant program within the last 2 years and a large proportion of participants were still in the process of donating. Survey participants were primarily Caucasian and included a larger proportion of women compared to men. Furthermore, male participants were older, more financially secure, while most women surveyed were of working age and in less-secure job situations. These demographics are somewhat reflective of the demographic characteristics among actual donors but may not be reflective of the larger pool of potential donors. Understanding barriers to donation amongst individuals who may have greater difficultly participating in English survey studies is critical. Specifically, understanding cultural and socioeconomic barriers to donation among women and men from racialized and marginalized populations is an important area for future research. Indeed, research initiatives, such as the A.C.T.I.O.N study,^37^ are utilizing culturally tailored qualitative methods to ensure participation of racialized donors and recipients when evaluating barriers to donation and transplantation.

## Conclusion

This study outlines important motivators and barriers for living kidney donation among individuals who underwent evaluation for living kidney donation and highlights the importance to more robustly address concerns relating to family, domestic, and financial considerations for potential donors. Importantly, the magnitude of concern was similar in both women and men, highlighting the need to address these issues in both groups.

## Supplemental Material

sj-pdf-1-cjk-10.1177_20543581221137179 – Supplemental material for Motivators and Barriers to Living Donor Kidney Transplant as Perceived by Past and Potential Donors Click here for additional data file. Supplemental material, sj-pdf-1-cjk-10.1177_20543581221137179 for Motivators and Barriers to Living Donor Kidney Transplant as Perceived by Past and Potential Donors by Julia Zazoulina, Keesha Khehra and Jagbir Gill in Canadian Journal of Kidney Health and Disease
