# Supplementary material for: Motivators and Barriers to Living Donor Kidney Transplant as Perceived by Past and Potential Donors
Source: Can J Kidney Health Dis. 2022 Nov 19;9:20543581221137179. doi: 10.1177/20543581221137179 (PMC9677302; doi:10.1177/20543581221137179)
Supplement: sj-pdf-1-cjk-10.1177_20543581221137179 – Supplemental material for Motivators and Barriers to Living Donor Kidney Transplant as Perceived by Past and Potential Donors [file sj-pdf-1-cjk-10.1177_20543581221137179.pdf]

Table 1. Study Cohort Demographics

	Total (N=	Gender	
		Male (N=	Female (N=
**Age Group**			
18-30	9 (6.5%)	4 (8.3%)	5 (5.6%)
31-40	15 (10.9%)	3 (6.3%)	12 (13.3%)
41-50	29 (21.0%)	5 (10.4%)	24 (26.7%)
51-60	34 (24.6%)	12 (25%)	22 (24.4%)
>60	49 (35.5%)	23 (47.9%)	26 (28.9%)
**Race**			
Caucasian	126 (91.3%)	43 (89.6%)	83 (92.2%)
Indigenous	3 (2.2%)	1 (2.1%)	2 (2.2%)
Asian	6 (4.3%)	3 (6.3%)	3 (3.3%)
Black	1 (0.7%)	0	1 (1.1%)
Middle Eastern	2 (1.4%)	1 (2.1%)	1 (1.1%)
**Born in Canada**			
Yes	117 (85.4%)	40 (83.3%)	77 (86.5%)
No	20 (14.6%)	8 (16.7%)	12 (13.5%)
**Relationship Status**			
In a relationship	106 (76.8%)	41 (85.4%)	65 (72.2%)
Single	18 (13.0%)	4 (8.3%)	14 (15.6%)
Divorced/Separated	13 (9.4%)	3 (6.3%)	10 (11.1%)
Widowed	1 (0.7%)	--	1 (1.1%)
**Sexual Orientation**			
Heterosexual	130 (94.2%)	44 (91.7%)	86 (95.6%)
LGBTQ	6 (4.3%)	3 (6.3%)	3 (3.3%)
**Education**			
Less than Highschool	3 (2.2%)	1 (2.1%)	2 (2.2%)
Highschool Diploma	15 (10.9%)	9 (18.8%)	6 (6.7%)
Some Post-Secondary	58 (42.3%)	22 (45.8%)	36 (40%)
Bachelor’s Degree	32 (23.4%)	7 (14.6%)	25 (27.8%)
Postgraduate Degree*	29 (21.2%)	9 (18.8%)	20 (22.2%)
**Current Employment**			
Working	93 (67.4%)	28 (58.3%)	65 (72.2%)
Retired	36 (26.1%)	18 (37.5%)	18 (20%)
Other**	9 (6.5%)	2 (4.2%)	7 (7.8%)
**Children**			
Yes	96 (69.6%)	32 (66.7%)	64 (71.1%)
No	42 (30.4%)	16 (33.3%)	26 (28.9%)
**Personal Income**			
<15K	34 (24.6%)	3 (6.3%)	8 (8.9%)
16-30K	19 (13.8%)	5 (10.4%)	14 (15.6%)
31-50K	32 (23.2%)	8 (16.7%)	24(26.7%)
51-75K	35 (25.4%)	18 (37.5%)	17 (18.9%)
76-90K	17 (12.3%)	4 (8.3%)	13 (14.4%)

>90K	23 (16.7)	10 (20.8%)	13 (14.4%)
**Household Income**			
<15K	2 (1.4%)	1 (2.1%)	1 (1.1%)
16-30K	5 (3.6%)	1 (2.1%)	4 (4.4%)
31-50K	23 (16.7%)	6 (12.5%)	17 (18.9%)
51-75K	34 (24.6%)	14 (29.2%)	20 (22.2%)
76-90K	21 (15.2%)	9 (18.8%)	12 (13.3%)
>90K	50 (36.2%)	17 (35.4%)	33 (36.7%)
Donation Status			
Potential donors			

** Includes Masters, Professional Degree, Doctorate*

*** Includes unemployed and looking for work, unemployed and not looking for work, and other*

Figure 1. Personal Income & Employment Industry

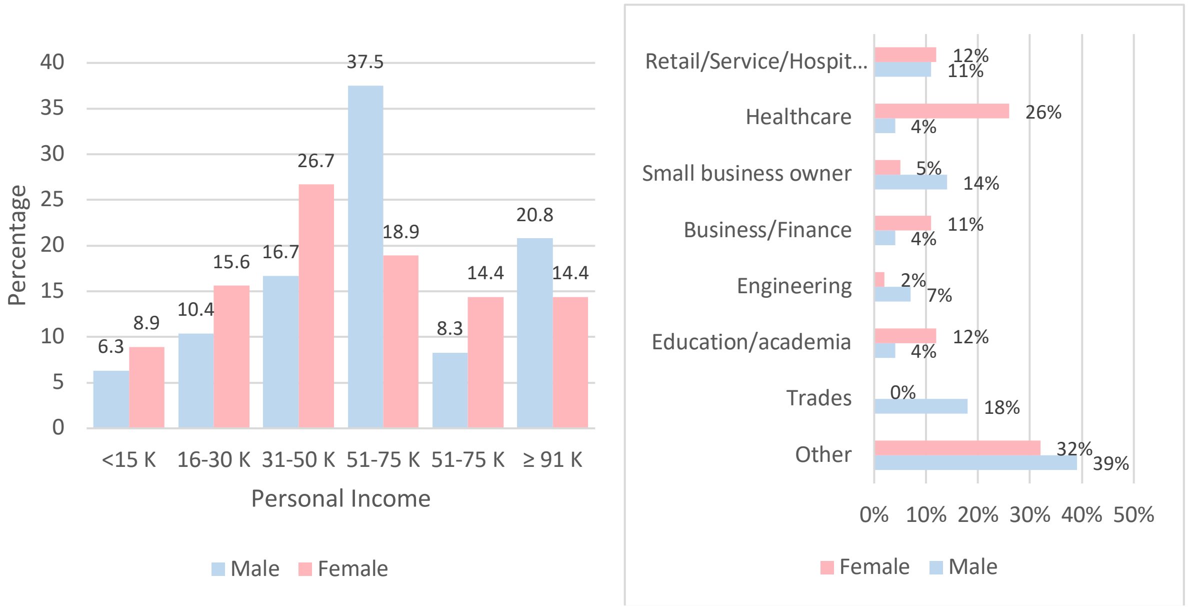

**Excluded individuals with no response for personal income (N=1)*

Figure 2A: Motivators and Barriers in relation to the Decision Making and Evaluation Process

Pink shaded bars represent female respondents and blue shaded bars represent male respondents.

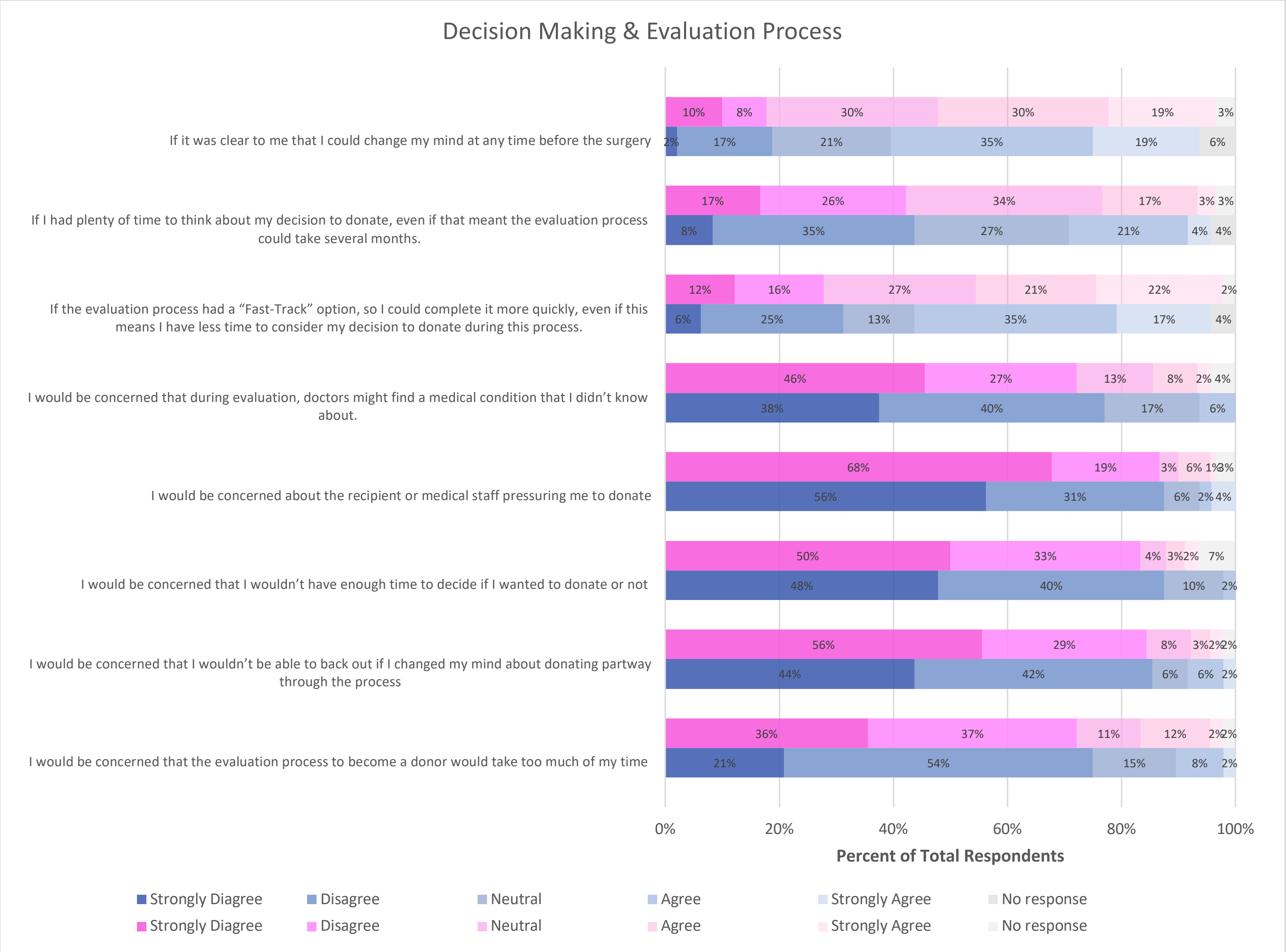

Figure 2B: Motivators and Barriers in relation to Family Pressures and Domestic Responsibilities

Pink shaded bars represent female respondents and blue shaded bars represent male respondents.

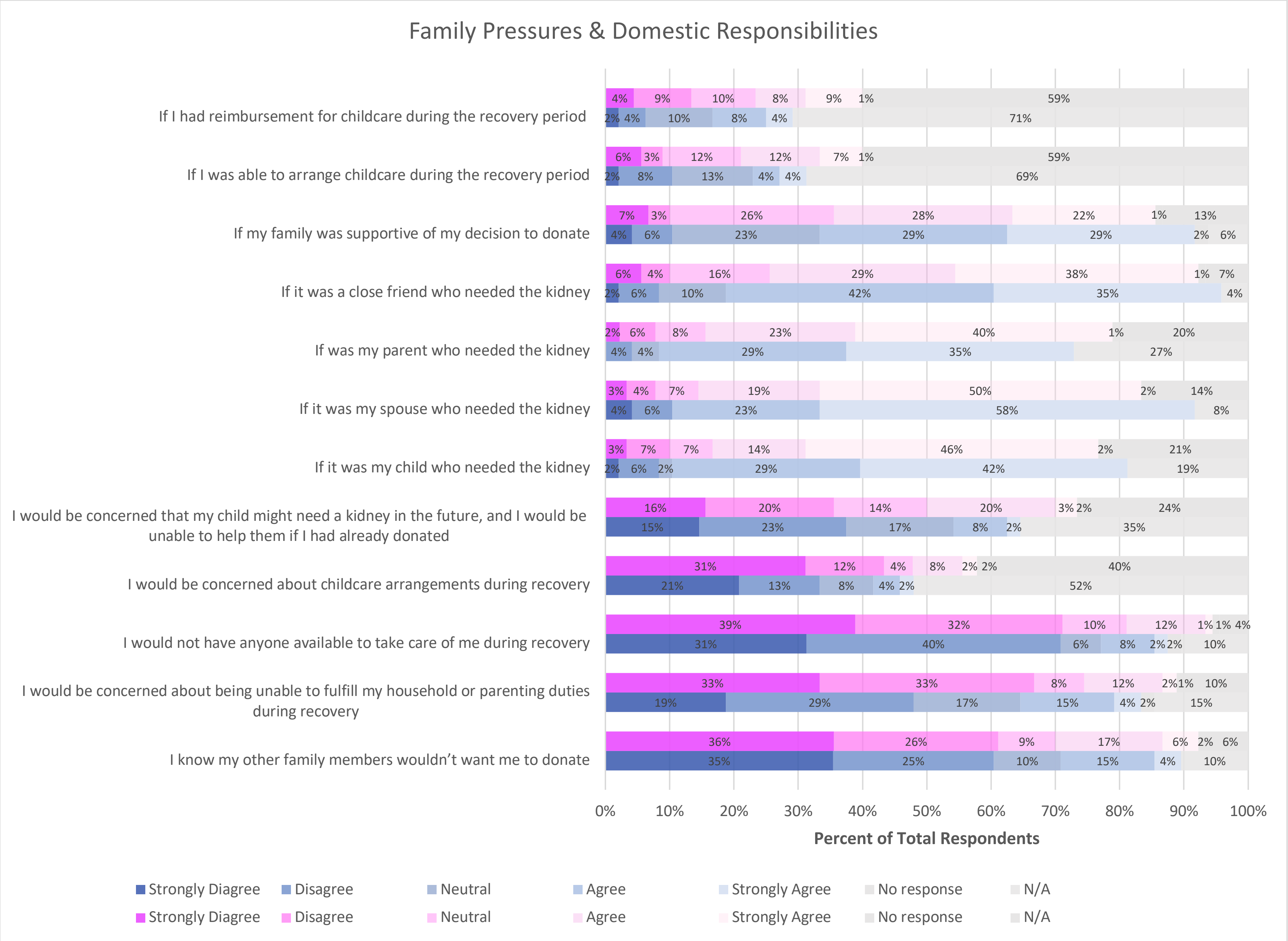

Figure 2C: Motivators and Barriers in relation to Finances and Job Security

Pink shaded bars represent female respondents and blue shaded bars represent male respondents.

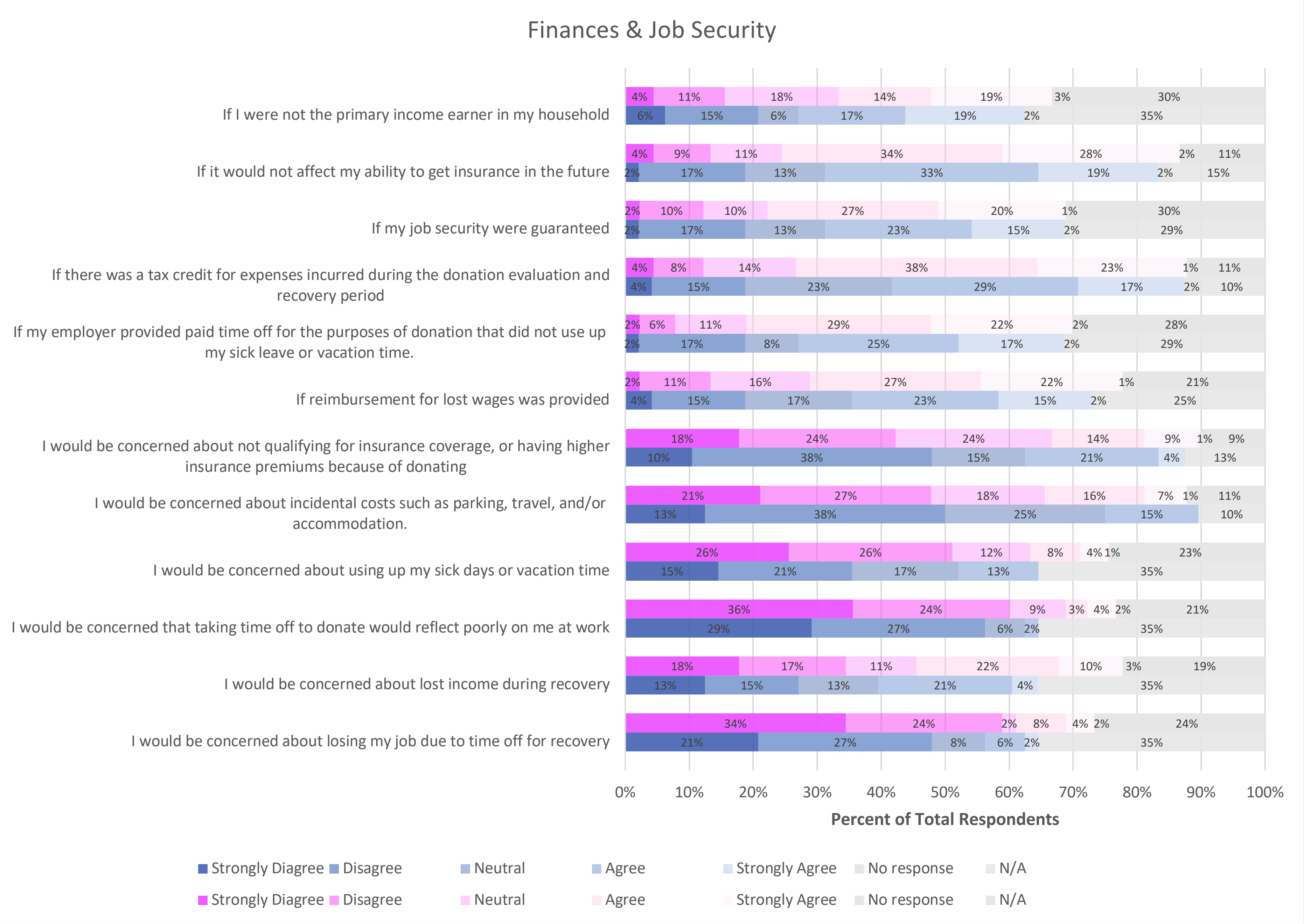

Figure 2D: Motivators and Barriers in relation to the Recovery Process

Pink shaded bars represent female respondents and blue shaded bars represent male respondents.

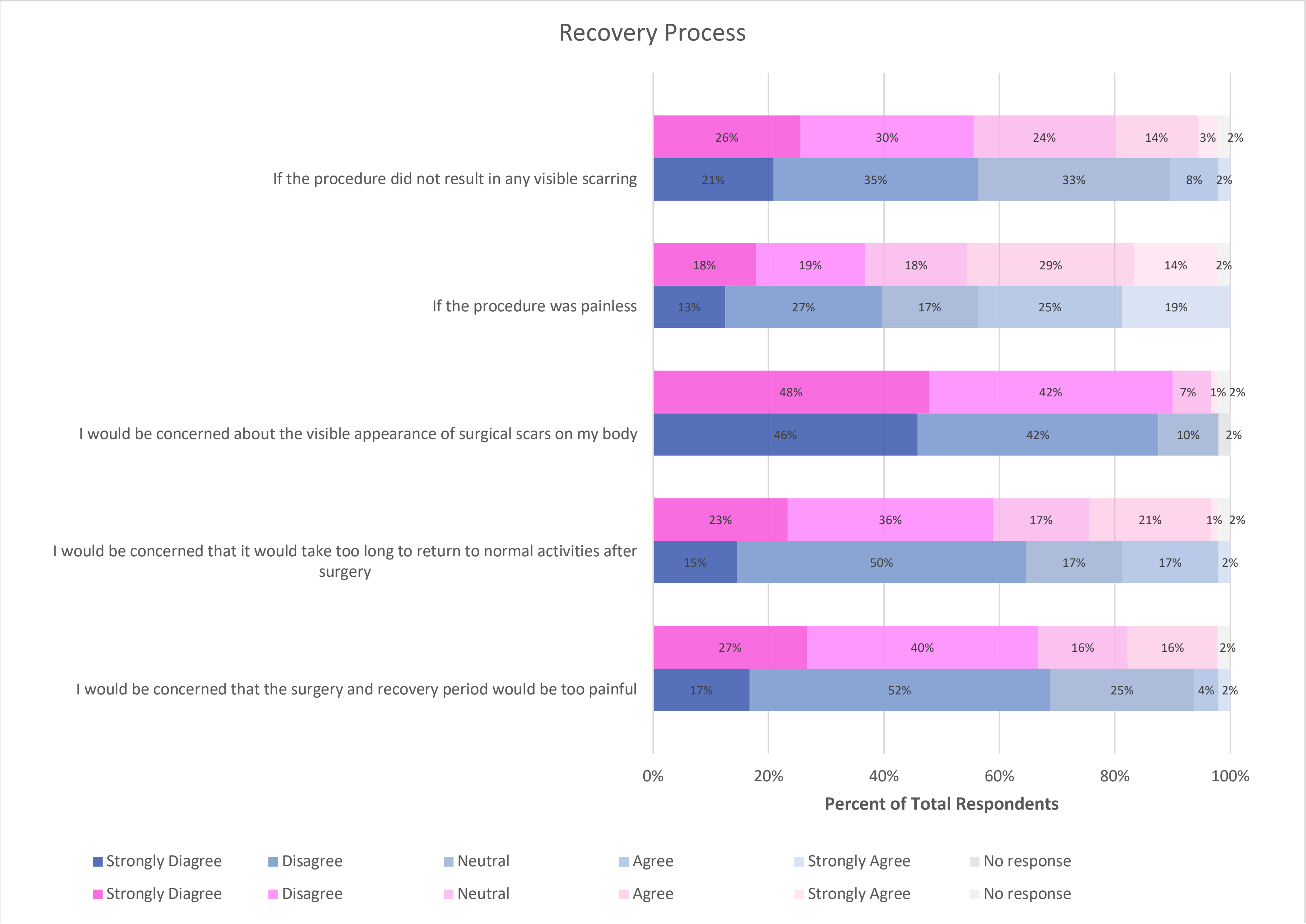

Figure 2E: Motivators and Barriers in relation to Complications

Pink shaded bars represent female respondents and blue shaded bars represent male respondents.

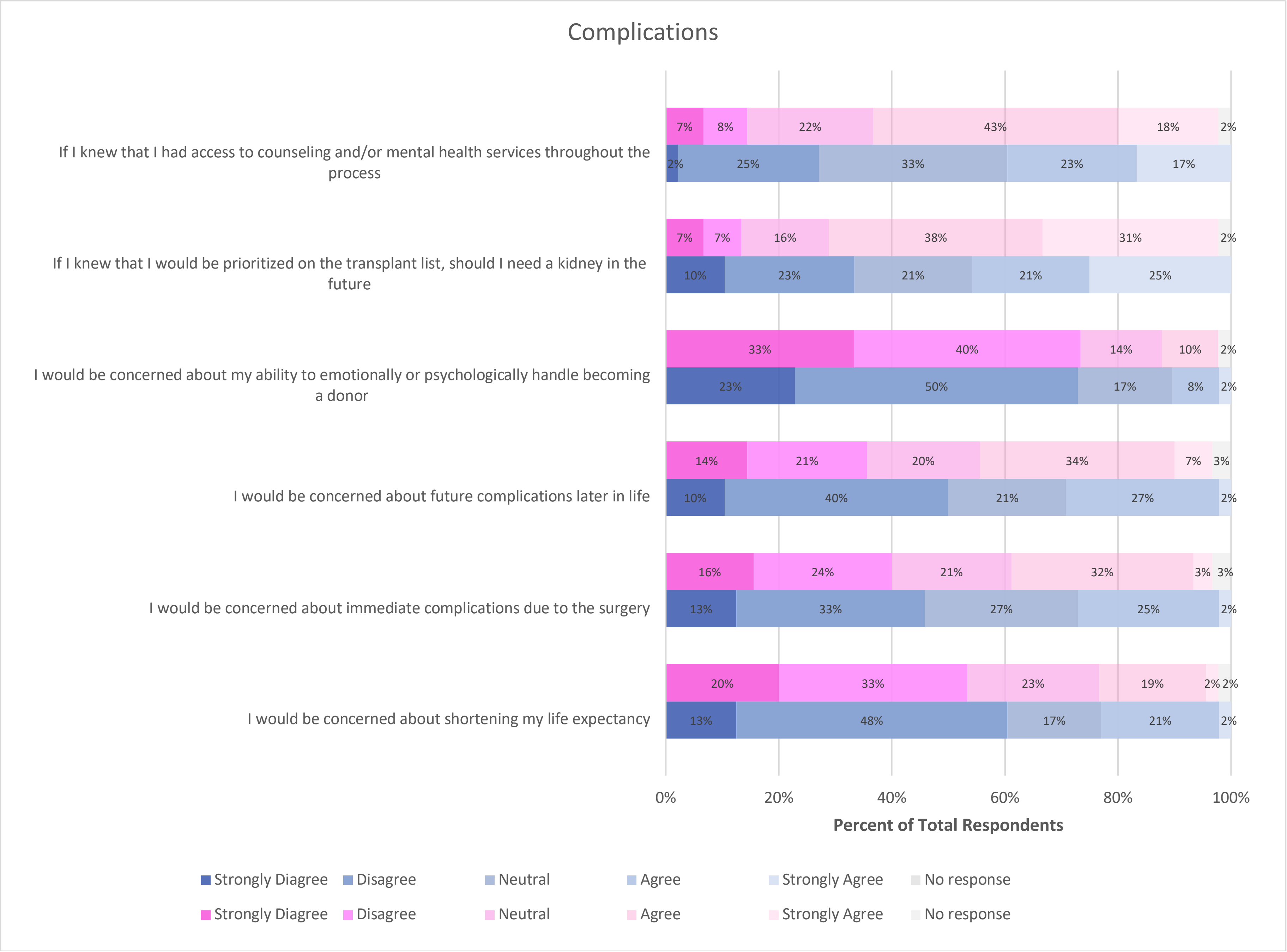

Figure 3. Median Barrier & Motivator Scores

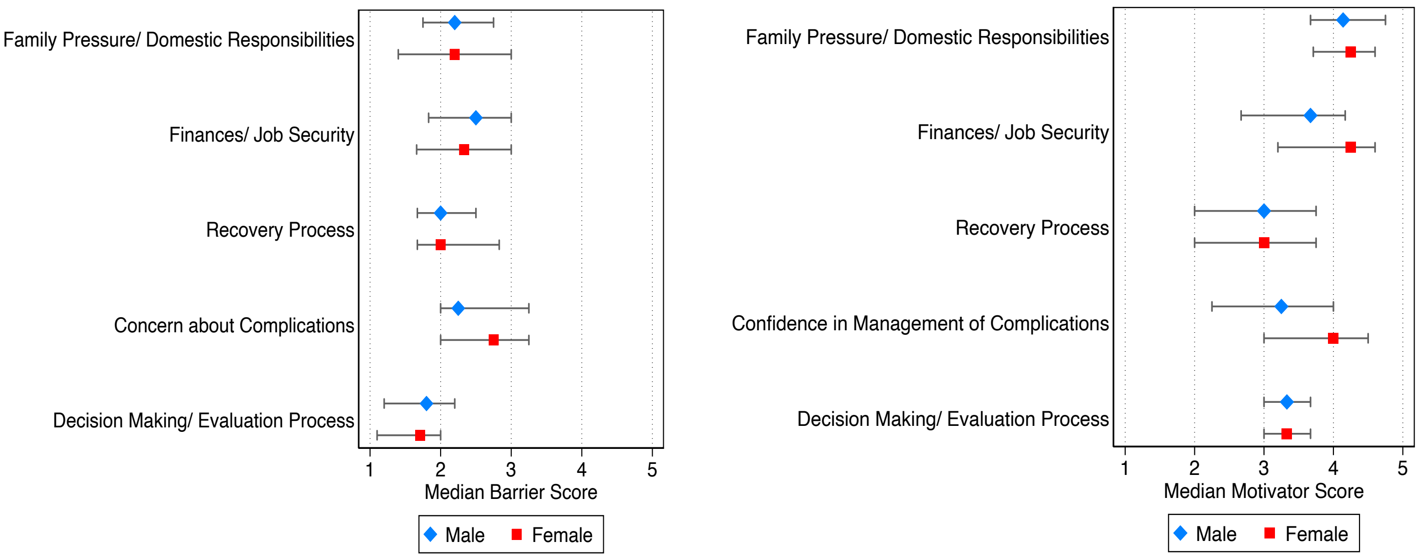

Supplementary Figure 1: Knowledge of Living Donor Kidney Transplant

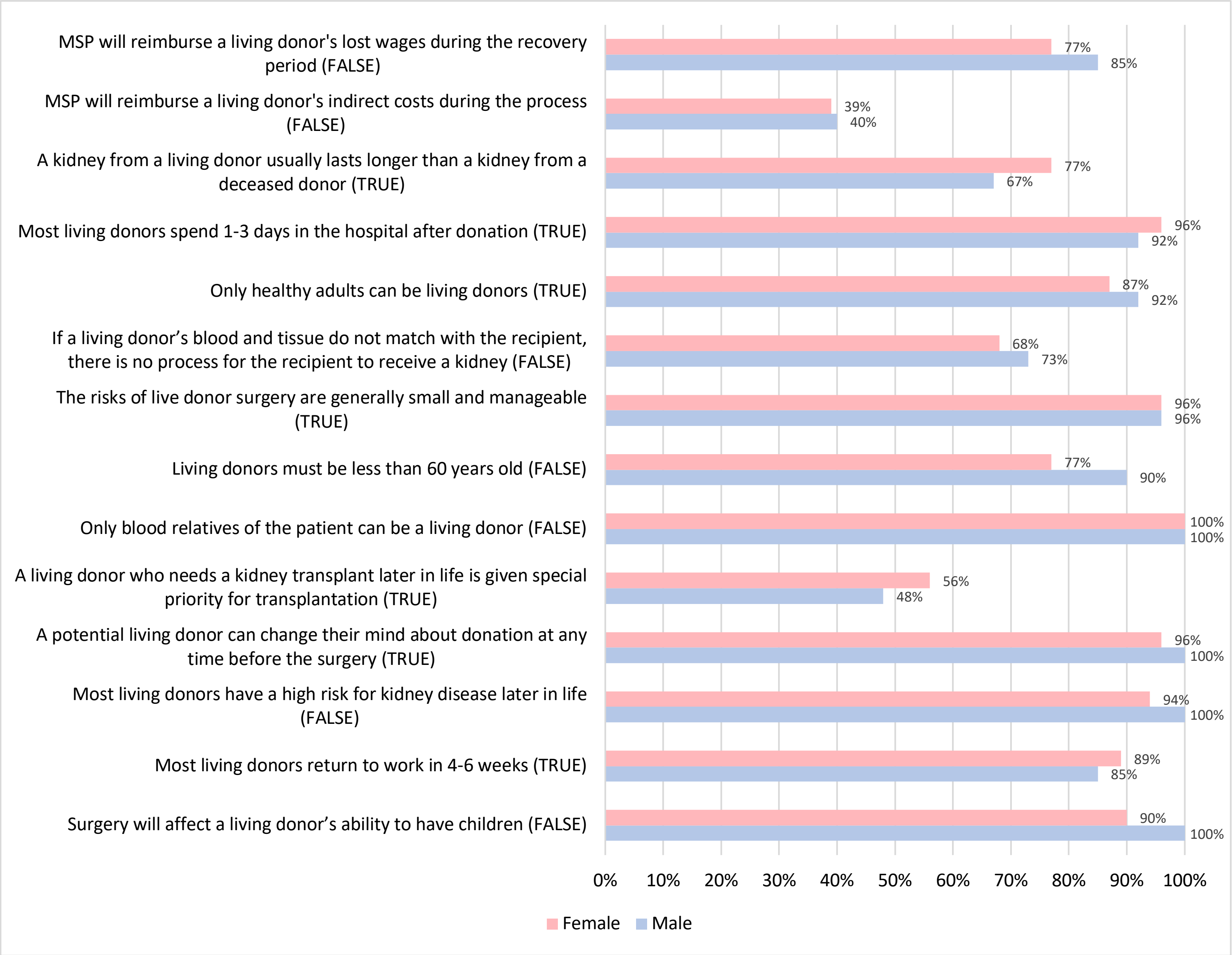

Supplementary Table 1: Barrier & Motivation Scores

Barrier Scores	Male		Female	
	**Median (Q2,Q3)**	**Mean**	**Median (Q2,Q3)**	**Mean**
Family Pressure/ Domestic Responsibilities	2.2 (1.75-2.75)	2.26	2.2 (1.4-3)	2.18
Finances/ Job Security	2.5 (1.83-3)	2.41	2.33 (1.66-3)	2.47
Recovery Process	2 (1.67-2.5)	2.10	2 (1.67-2.83)	2.07
Complications: Concern about Complications	2.25 (2-3.25)	2.53	2.75 (2-3.25)	2.57
Decision Making/ Evaluation Process	1.8 (1.2-2.2)	1.85	1.71 (1.1-2)	1.76

Motivation Scores	Male		Female	
	**Median (Q2,Q3)**	**Mean**	**Median (Q2,Q3)**	**Mean**
Family Pressure/ Domestic Responsibilities	4.14 (3.67-4.75)	4.01	4.25 (3.71-4.6)	3.96
Finances/ Job Security	3.67 (2.67-4.17)	3.40	4 (3.2-4.5)	3.77
Recovery Process	3 (2-3.75)	2.73	3 (2-3.75)	2.71
Complications: Confidence in Management in Complications	3.25 (2.25-4)	3.27	4 (3-4.5)	3.70
Decision Making/ Evaluation Process	3.33 (3-3.67)	3.21	3.33 (3-3.67)	3.11
